# 11.2 THE IMPACT OF AEROBIC EXERCISE ON COGNITIVE FUNCTIONING AND BIOMARKERS OF COGNITIVE CHANGE IN INDIVIDUALS WITH SCHIZOPHRENIA

**DOI:** 10.1093/schbul/sby014.040

**Published:** 2018-04-01

**Authors:** David Kimhy, Julia Vakhrusheva, Matthew Bartels, Jacob Ballon, Eero Castrén, Richard Sloan

**Affiliations:** 1 Icahn School of Medicine at Mount Sinai; 2 Columbia University; 3 Albert Einstein College of Medicine; 4 Stanford University; 5 University of Helsinki

## Abstract

**Background:**

Individuals with schizophrenia (SZ) display substantial cognitive deficits across multiple domains. These deficits have been identified as major determinants of poor functioning and disability, representing a serious public health concern and an important target for interventions. At present, available treatments offer only minimal to limited benefits to ameliorate these deficits. Thus, there remains an urgent need to identify novel treatments for cognitive deficits in people with SZ. Emerging evidence from studies of animals, clinical and non-clinical populations suggest that Aerobic Exercise (AE) is efficacious in improving cognition via up-regulation of Brain-Derived Neurotrophic Factor (BDNF). Yet, the impact of AE on cognition and daily-functioning, and the role of BDNF, have not been investigated in schizophrenia. Additionally, limited information is available on the putative link between inflammation markers to cognitive functioning.

**Methods:**

Employing a single-blind RCT design, 33 individuals with schizophrenia were randomized to receive “treatment as usual” (n=17; TAU) or attend a 12-week, 3 times-per-week, 60-minutes AE program (n=16) utilizing active-play video-games (Xbox-360 Kinect) and traditional AE equipment.

**Results:**

At baseline, cognitive functioning was associated with serum BDNF (r=.51, p=.01), along with TNF-alpha (r=-.38, p=.03), IL-12 (r=-.36, p=.04) and IL-6 (r=-.33, p=.06). Twenty-six participants completed the study (79%). Following the intervention, the AE participants improved their cognitive functioning (MCCB) by 15.1% (vs. -2.0% in the TAU group; p=.03). Hierarchical multiple-regression analyses indicated changes in AF and serum BDNF predicted 25.4% and 14.6% of the cognitive improvement, respectively. Additionally, changes in aerobic fitness (VO2peak ml/kg/min) correlated with informant-reported improvements in work-related daily-functioning skills (SLOF; r=.51, p=.01). Fidelity with target training intensity, was correlated with cognitive improvement (r=.70, p=.02).

**Discussion:**

The results indicate AE is effective in enhancing cognitive and daily functioning skills in people with schizophrenia and provide support for the impact of AE-related BDNF up-regulation on cognition. Additional studies are needed to establish the link between inflammation markers and cognitive functioning and the potential impact of AE on this putative pathway. Overall, low aerobic fitness represents a modifiable risk-factor for cognitive dysfunction in schizophrenia for which AE training offer a relatively safe, non-stigmatizing, and side-effect-free intervention.

